# A Vehicle-Edge-Cloud Framework for Computational Analysis of a Fine-Tuned Deep Learning Model

**DOI:** 10.3390/s24072080

**Published:** 2024-03-25

**Authors:** M. Jalal Khan, Manzoor Ahmed Khan, Sherzod Turaev, Sumbal Malik, Hesham El-Sayed, Farman Ullah

**Affiliations:** 1 College of Information Technology, United Arab Emirates University, Abu Dhabi 15551, United Arab Emirates; 201990067@uaeu.ac.ae (M.J.K.); manzoor-khan@uaeu.ac.ae (M.A.K.); 201990107@uaeu.ac.ae (S.M.); helsayed@uaeu.ac.ae (H.E.-S.); farman@uaeu.ac.ae (F.U.); 2 Emirates Center for Mobility Research (ECMR), United Arab Emirates University, Abu Dhabi 15551, United Arab Emirates

**Keywords:** autonomous vehicles, deep learning, object detection, transportation

## Abstract

The cooperative, connected, and automated mobility (CCAM) infrastructure plays a key role in understanding and enhancing the environmental perception of autonomous vehicles (AVs) driving in complex urban settings. However, the deployment of CCAM infrastructure necessitates the efficient selection of the computational processing layer and deployment of machine learning (ML) and deep learning (DL) models to achieve greater performance of AVs in complex urban environments. In this paper, we propose a computational framework and analyze the effectiveness of a custom-trained DL model (YOLOv8) when deployed in diverse devices and settings at the vehicle-edge-cloud-layered architecture. Our main focus is to understand the interplay and relationship between the DL model’s accuracy and execution time during deployment at the layered framework. Therefore, we investigate the trade-offs between accuracy and time by the deployment process of the YOLOv8 model over each layer of the computational framework. We consider the CCAM infrastructures, i.e., sensory devices, computation, and communication at each layer. The findings reveal that the performance metrics results (e.g., 0.842 mAP@0.5) of deployed DL models remain consistent regardless of the device type across any layer of the framework. However, we observe that inference times for object detection tasks tend to decrease when the DL model is subjected to different environmental conditions. For instance, the Jetson AGX (non-GPU) outperforms the Raspberry Pi (non-GPU) by reducing inference time by 72%, whereas the Jetson AGX Xavier (GPU) outperforms the Jetson AGX ARMv8 (non-GPU) by reducing inference time by 90%. A complete average time comparison analysis for the transfer time, preprocess time, and total time of devices Apple M2 Max, Intel Xeon, Tesla T4, NVIDIA A100, Tesla V100, etc., is provided in the paper. Our findings direct the researchers and practitioners to select the most appropriate device type and environment for the deployment of DL models required for production.

## 1. Introduction

The transition from traditional to smart roads for realizing an intelligent environment and enabling autonomous driving (AD) requires a significant shift in how we approach transportation infrastructure. The conceptual canvas of smart roads represents an integration of autonomous vehicles (AVs), evolved roadside units (eRSUs), and central data centers to improve safety, efficiency, and mobility [1,2]. For instance, the cooperative connected and automated mobility (CCAM) infrastructure creates an extended perception of the road segments [3]. Similarly, we need artificial intelligence (AI) mechanisms for different decision-making instances to enable AVs to perform critical and non-critical operations during driving. Although it is imperative to deploy trained machine learning (ML) and deep learning (DL) models at CCAM infrastructure, it is also necessary to adhere to deployment instructions and processes when the models are deployed for perception tasks, i.e., object classification, object detection, object tracking, object segmentation, object prediction, etc., by the AVs. Moreover, the deployed DL models allow AVs to plan and update their trajectories, execute acceptable actions, and perform controllable operations. Therefore, it is highly recommended to deploy a DL model, which is most relevant to the underlying use case scenarios to maintain the high performance of the AVs.

The existing research works have explored a spectrum of solution approaches and proposed a plethora of DL-based models for conducting perception tasks in AD, ranging from single-modal to comprehensive multi-modal perception systems [1,4]. The KITTI [5] and nuScenes [6] datasets have been instrumental in producing benchmark methods [7,8] for perceptual operations of AVs. This means that the relevant academic community dedicated to this field is rapidly developing systems to enhance AD perception, leveraging real-world data and leading-edge datasets such as nuScenes, KITTI, and Waymo [9]. For instance, these systems include SparseFusion [10], CMT-NaiveDETR [11], CBM-Fusion [12], MSMDFusion-TA [13], Adaptive-Fusion [14], Inter-Frame Fusion [15], etc. These systems are applied in crucial perceptual tasks like object recognition, motion tracking, and navigational planning. In addition, a selective number of studies e.g., DCNN-Fusion [16], E-DCNN [17], WM-YOLO [18], etc., have introduced methods to address challenges like feature mismatches and omissions in object detection to refine the accuracy of perception. Furthermore, a lightweight YOLOv8-CB [19] enhanced pedestrian detection accuracy. The system [20] improved the visual capabilities using YOLO-based object detection on edge computing. In [21], the authors performed object detection on Jetson AGX Xavier in edge computing. The TF-YOLO detector [22] enhanced pedestrian detection with a novel transformer–fusion module. The IDLVD-UARSI technique [23] achieved high accuracy in vehicle detection using remote sensing imagery. In [24], the authors proposed a DL-based system for face recognition in CCTV images, aiming for high accuracy with minimal human oversight. In [25], the authors installed a smart camera using YOLOv7-tiny and Deep SORT on Nvidia Jetson Nano to improve traffic management by detecting vehicles.

Considering the existing literature and research studies, the processes for computing and deploying DL-based models across diverse devices in various environments for AVs using CCAM infrastructure have yet to be fully developed. In this research, we introduce an innovative computational framework aiming to evaluate DL model deployments and analyze temporal performance on a range of device types. The motivation for this research centers on enhancing the performance of AVs in complex urban environments through the CCAM infrastructure. Specifically, we focus on the efficient deployment of fine-tuned DL models within CCAM infrastructure, aiming to understand how the accuracy and execution time of DL models interact and affect the performance when deployed over various devices across different layers, i.e., vehicle, edge, and cloud of the proposed framework. Devices such as Raspberry Pi 4 [26], Jetson AGX Xavier [27], Apple M2 Max [28], NVIDIA V100, Tesla A100, etc., are equipped with and without GPUs using the CCAM infrastructure at different layers of the framework. Some of the major contributions of this research work are the following:

- The design and development of an innovative computational framework comprised of vehicle, edge, and cloud layers inspired by CCAM infrastructure.
- Acquisition of a novel custom-formatted multi-object dataset for perception tasks.
- Deployment of the fine-tuned DL YOLOv8 model over various devices, i.e., Raspberry Pi 4, Jetson AGX ARMv8, Jetson AGX Xavier, Apple M2 Max, Apple MPS, Intel Xeon CPU, Tesla T4, NVIDIA A100, and Tesla V100.
- Performance evaluation through metric-based analysis and comparative assessment of average times for various stages of perception task.

The paper is organized into five sections. Section 2 presents an overview of the proposed framework, data acquisition, fine-tuning approach, and different layers of the framework. Section 3 presents the experimental setup, evaluation metrics, and experiments. Section 4 presents the results and discusses the model performance and time sensitivity comparison. Section 5 concludes the paper.

## 2. An Overview of the Proposed Framework

In the conceptual canvas of smart roads, the AVs utilize AD technology to perform safe navigation and driving. All thanks to the enhancements provided by infrastructures like CCAM [3], which enrich AV perception and contextual understanding, and support decision-making operations within AVs. It should be highlighted that the motivation behind contributing to the proposed computational framework is driven by CCAM infrastructure and the need to develop and deploy solutions at different levels for applying AD technology in complex urban environments. Therefore, this section is dedicated to the design and detailed description of the proposed computational framework as well as the necessary infrastructures of CCAM, i.e., sensory devices, communication, and computation at each layer of the proposed framework. It should also be highlighted that the proposed computational framework draws from the authors’ practical experience in implementing these concepts in both Europe and the UAE. Therefore, the readers are directed to [1,2,3] for further engagement in these activities.

**Figure 1:**
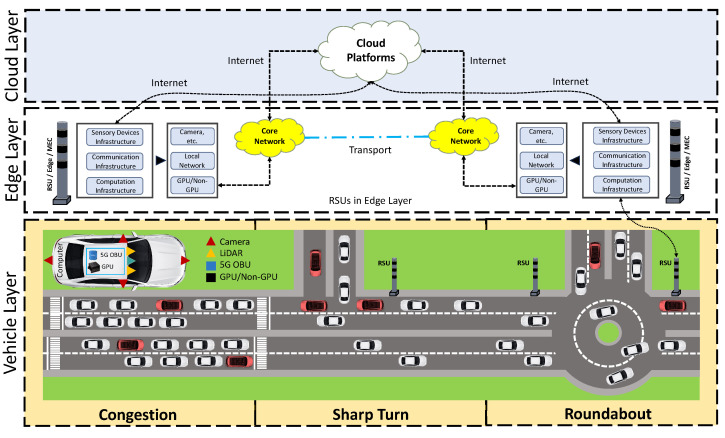
The proposed computational framework for deep learning model deployments.

To handle the dynamic maneuvering of AVs, the ML and DL-based algorithms and models need to be efficient and accurate. Since the AVs need DL-based models for different decision-making instances during driving, we present an abstract-level view of the DL models’ deployment process at the three-layer solution framework to facilitate AVs, as shown in Figure 1. To understand the solution framework, let us provide a brief overview of the DL model deployment at three layers, i.e., vehicle layer, edge (roadside) layer, and cloud (central data-center) layer, for AD at the three different use case scenarios, i.e., congestion, sharp turn, and roundabout at UAE University, UAE. The solution framework includes infrastructures, i.e., sensory devices, communication, and computations at each layer. Furthermore, the framework also includes a gateway (IoT middleware) and middleware APIs. Similarly, at each layer, the capturing devices in sensory infrastructure are used to capture data, the network devices and IoT middleware in communication infrastructure are used to make the data available to DL-based models, the DL models (resting in a smart decision engine) deployed at devices in the computation infrastructure are used to perform perception tasks (e.g., object detection, etc.), and the inferences of the perception tasks (e.g., detected objects) are used to help in decision-making of the AVs. Now that we have our three-layer solution framework, we capture data from various complex urban road segments.

### 2.1. Data Acquisition and Model Fine-Tuning

**Figure 2:**
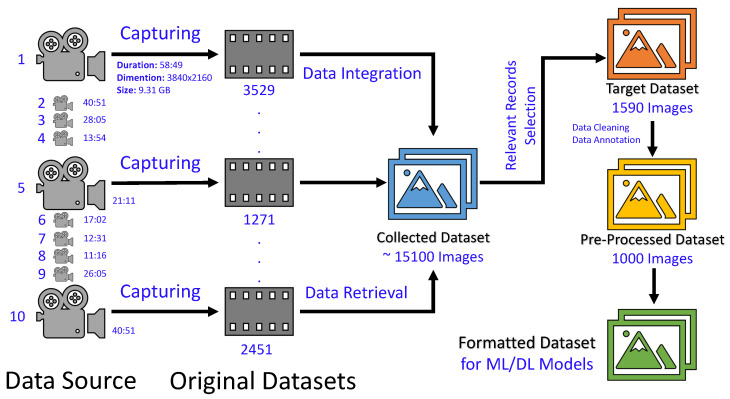
The proposed pipeline for data preparation.

The data are captured in the form of video clips (data sources) using a high-resolution camera mounted on a vehicle with a rate of 60fps (frames per second) such that the resolution is 3840 × 2160. During the data collection process, we captured 14 different video clips of varying lengths. Figure 2 displays a selection of 10 of these clips, detailing their durations and illustrating the comprehensive dataset pipeline used for data preparation. With the data sources in place, we utilized the widely recognized FFmpeg [29] tool to extract spatial data records, specifically images, from these sources. The sizes of the original datasets varied due to the differences in the size and duration of the data sources. Subsequently, we merged these original datasets by collating essential data details, thereby creating a unified dataset. Consequently, our compiled image dataset is comprised of 15,100 images, where the main objects are cars, persons, motorcycles, and trucks. For data pre-processing, we rely on data sampling and reduce data volume by focusing on longer-term changes. Consequently, we obtained 1590 images that were further filtered through data cleaning and resulted in 1000 images. These images were considered for manual labeling as some perception tasks (e.g., object detection, etc) require objects along their label and classes. The dataset consists of five different classes, i.e., cars, pedestrians, motorcyclists, trucks, and rickshaws. For the DL model, we rely on a well-known split ratio of 70:20:10, which means 70% data for training, 20% data for validating, and 10% data for testing. It should be highlighted that we used the same target unseen test dataset for each device of the computation infrastructure at each layer of the framework. In the following, we provide the details for the perception task, DL model selection, and hyperparameter fine-tuning.

**Figure 3:**
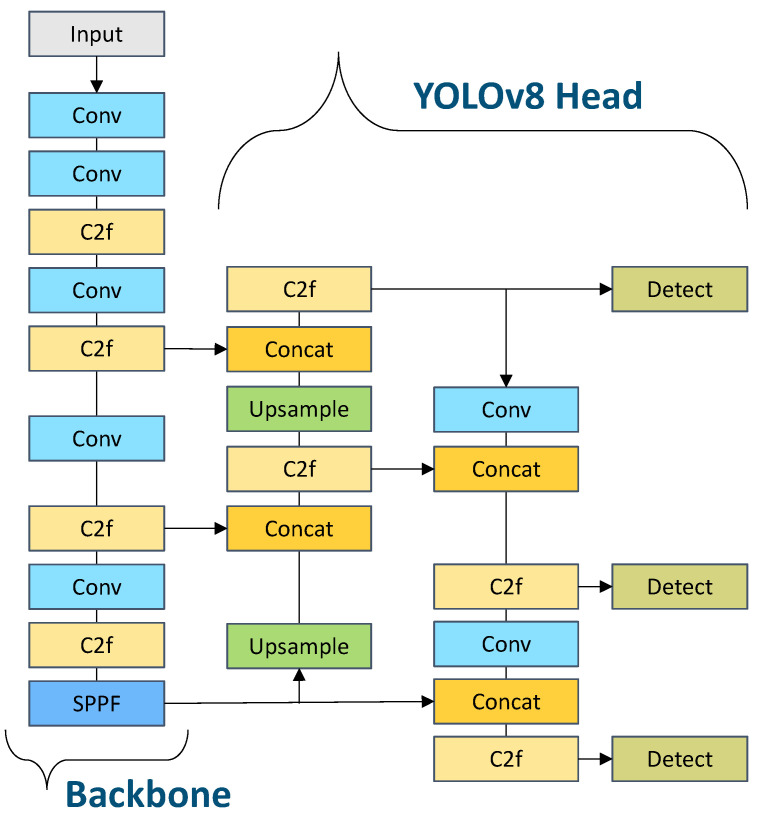
The state-of-the-art deep learning YOLOv8 model architecture.

In AVs, the perception system performs many different tasks such as object detection, tracking, segmentation, and prediction. In object detection, the object detector’s decision is based on the deep-layered learning of the model. However, the selection of a DL model is not easy and mostly depends on the underlying use case scenarios [4]. In this research work, we choose the state-of-the-art (SOTA) DL-based You Only Look Once (YOLOv8) model [30] to perform object detection. The YOLO family has a huge history and YOLOv8 (released on 10 January 2023) comes with five different versions. The architecture of YOLOv8 is shown in Figure 3. Moreover, it has been used for traffic object detection [31] in traffic environments [32]. Now that we have our pre-processed dataset and DL-based YOLOv8 model, we perform the benchmarking of different instances of the model by training and hyperparameter tuning. In this connection, we consider the following hyperparameters: SGD, Adam, RMSProp, and AdamW, categorized as *optimizers*; 16 and 32, categorized as *batch sizes*; and 100, 150, and 200, categorized as *epochs*. The configuration of the AdamW-based YOLOv8 model outperforms all other configurations. Therefore, we select the AdamW optimizer, a batch size of 32, and 200 epochs as the optimal configuration for our custom-trained fine-tuned YOLOv8s model for the object detection-based perception task. Now that we have our fine-tuned DL-based YOLOv8s model, we start deploying it at each layer of the proposed computational framework.

### 2.2. The Vehicle Layer

The vehicle layer comprises several key components that enable the AV to navigate roads by capturing the environment, which is crucial for the perception tasks. The following are three key components of the vehicle layer. In the sensory infrastructure, our starting point is the camera sensor, although options like LiDAR, radar, and other sensory devices are also available. We use the camera sensor to capture the objects over the road. The spatial data are recorded at 30 fps; therefore, the capture time for every image on average is equal to 34.34 ms. It is important to highlight that each sensory device captures the road data in a different format. In the communication infrastructure, dedicated short-range communication (DSRC) and cellular technology (LTE-5G) are used to transmit (receive) captured spatial data to (from) the edge layer. It is worth noting that if a computationally powerful gateway device, e.g., Jetson Xavier AGX, etc., is available at AV, then there is no need to transmit the data for inferencing to the edge layer. In our use case, we bypass the communication infrastructure when the data are immediately available to the deployed DL-based YOLOv8s model on the same device. However, we utilize the communication infrastructure when transmitting spatial data to the Google Cloud via the Ethernet interface for another deployed DL YOLOvs model at the edge layer and cloud layer. In the computation layer, we focus on two different types of devices: those with and without GPU capabilities, i.e., Jetson AGX and Raspberry Pi 4. It is important to highlight that Jetson AGX comes with both GPU and non-GPU capabilities; therefore, we use both available options. However, Raspberry Pi 4 (RPi) only comes with non-GPU capabilities. Hence, these devices make three different platforms for the DL model deployment. These devices are also considered gateway devices for the vehicle, performing upstream operations to provide drivable environmental scenes (spatial data) to the edge layer and cloud layer.

Now that we have infrastructures in the framework, we deploy our custom-trained DL-based YOLOv8s model on Jetson (GPU) and RPi (non-GPU) devices to facilitate the object detection task for the vehicle’s perception system. The computation devices will leverage the trained YOLOv8s model to detect objects in the road segment, creating a perception of the road. The created perception of the road is then transferred to the vehicle. For instance, a pedestrian is moving on a road segment. The vehicle will use the provided perception and depending on the driving condition and road segment in that specific region, it performs the object detection task and provides inference. Based on the inference, the vehicle will perform informed decision-making. The vehicle decision to utilize perception from the vehicle layer (onboard perception system) or edge layer (on-road perception system) depends on various factors, including the complexity of the environment, the desired level of redundancy and robustness, and the specific use case or application of the autonomous vehicle. In some scenarios, such as highly dynamic or congested urban environments, the additional perception data from the edge layer can provide external information and enhance the overall perception capabilities of the vehicle. Now that we have a working principle at the vehicle layer, we need to see if the vehicle transfers the capture data to the nearest edge server. In the following, we discuss the edge layer.

### 2.3. The Edge Layer

The second layer of our proposed computational framework is the edge layer, which includes similar key components. The eRSUs of the edge layer play a crucial role in enabling AD technology. In the sensory infrastructure, we have the option to use and mount various sensors, i.e., camera, LiDAR, radar, etc. to capture data of a road segment in a specific region. At the edge layer, the eRSU creates a perception of a road segment or a digital twin of a region. This means that the sensory data captured on eRSUs is used to create a robust perception system for AVs and the required ingredients to create perception through the sensory data rely on the types of sensors. It is important to highlight that a perception system through sensory data can be created at both the vehicle layer and edge layer. Moreover, the captured spatial records can also be transferred (received) to (from) the upper and lower layers of the framework. In this research, we rely on the data captured by the camera device in the sensory infrastructure at the vehicle layer. Therefore, we do not capture data directly at this layer. The main reason behind this approach is to provide a fair comparison to the deployed custom-trained DL-based YOLOv8s models by providing the same dataset. In the communication infrastructure, the sensory devices are connected through switches, routers, Ethernet (local network), etc., and the communication is performed through either the core network or the Internet. The LTE and 5G cellular technologies are used to receive captured data from the vehicle layer. However, if the spatial data are captured at the edge layer, then these communication technologies forward the captured data to the vehicle and cloud layers. This means the eRSUs in the edge layer transmit the raw sensory data of the road segments to AVs and the data centers in the cloud.

We maintain the same approach in the computation infrastructure by using two different types of devices: GPU-based and non-GPU-based for computational processes and operations. To gain a fair comparison for the deployed DL-based YOLOv8s models, we select a single computational device that has both GPU and non-GPU capabilities. Moreover, we avoid using the Jetson AGX at the edge layer for similar reasons. Therefore, we select the Apple M2 MAX as the edge server because it has both GPU and non-GPU capabilities. Now that we have infrastructures at the edge layer, we deploy the custom-trained YOLOv8s model at the edge server. The edge server receives the spatial data from the vehicle layer and leverages the trained YOLOv8s model deployed at the edge layer to perform perception tasks, i.e., object detection. Once the objects are detected, the inferences are utilized within the edge layer and shared with the vehicles at the vehicle layer. This means that the eRSU creates a perception of the road in the form of object detection and the inference of object detection is provided to the AVs depending on the underlying use case scenarios, i.e., whether the AVs want to use their own perception system or would like to use eRSU’s perception system. Now that we have a working principle at the edge layer, we move to the cloud layer and describe its role in the overall framework. In the following, we discuss the cloud layer of the framework.

### 2.4. The Cloud Layer

The third and last layer of our computational framework is referred to as the cloud layer. The operations are offloaded to the cloud layer if the vehicle layer and edge layer are occupied or if the underlying use-case scenarios require substantial computational resources for intensive critical and non-critical applications. A sensory infrastructure is not necessary since we do not need to perform data capturing at this layer. However, if there is a specific requirement, i.e., monitoring, etc., then the data can be utilized for analysis and processing. When the cloud servers (central data centers) receive the spatial data, they start data pre-processing operations (in the case of raw data). It is important to highlight that we apply data mining techniques to perform data sampling and pre-processing to make data usable for the deployed DL-based YOLOv8s models. Once the spatial data are ready at the cloud layer, the data center transmits the processed sensory data to the eRSUs of the edge layer, which further share them with AVs to help in their decision-making activities. Here, we describe how to utilize the external perception created by the eRSU in the edge layer. The communication is performed through the internet. The external perception of the targeted road segment is created and can be transmitted to AVs for critical and non-critical applications, for instance, the applications for avoiding collisions, emergency situations, advanced warnings, etc.

It is worth mentioning that the success of such applications is incomplete without the use and application of DL-based algorithms and models. In this connection, we have deployed custom-trained DL-based YOLOv8s models for the object detection task of the perception system. We continue with the same approach in the computation infrastructure by using two different types of devices: GPU-based and non-GPU-based for computational processes and operations. The main reason behind this approach is to provide a fair comparison of the deployed YOLOv8s models and provide a holistic approach to computational comparison. In this connection, we select the Google platform since it has both GPU and non-GPU devices and fulfills our requirements. From the Google platform, we select Intel Xeon as a non-GPU-based device and NVIDIA Tesla V100, NVIDIA A100, and Tesla T4 as GPU-based devices. Using these computationally intensive devices, the cloud layer enables different critical and non-critical applications for AD technology. Now that we have detailed our proposed framework, we will discuss the experimental setup for the object detection-based perception task and the results of the fine-tuned DL-based YOLOv8 model deployed across various device types at each layer of the computational framework.

## 3. Experiments

We performed experiments using the custom-trained fine-tuned YOLOv8s DL model and deployed it over various devices across each layer of the proposed computational framework. For the device type, we considered GPU and non-GPU-based devices at the vehicle layer, edge layer, and cloud layer. In the following, we will provide details for our experimental setup, evaluation metrics, and results.

### 3.1. Experimental Setup

The experiments and deployments were carried out at each layer of the proposed framework. A complete list of platforms, devices, device types, and memory against each layer is provided in Table 1. At the vehicle layer, we deployed the fine-tuned YOLOv8s model over Raspberry Pi 4 and Jetson AGX devices. At the edge layer, we deployed the fine-tuned YOLOv8s model over Apple M2 Max devices. At the cloud layer, we deployed the fine-tuned YOLOv8s model over different devices from Google Cloud accessed through Google Colab Pro platform. The cloud layer devices were accessed through Google Chrome (version 117.0) installed in a physical machine of Intel Quad-Core i7@ 3.4 GHz with 32 GB NVIDIA GeForce GTX 680MX Graphics and a 27-inch (2560 × 1440) build-in Display. The software stack included Python v.3.11.4 (the newest major release), and its rich family of libraries, e.g., PyTorch, glob, etc. The DL-based YOLOv8s models were implemented through the *Ultralytics* package [33] using the latest version of Python programming language. The DL models were trained on an image size of 640 × 640, and different configurations were applied during the hyperparameter fine-tuning stage.

### 3.2. Evaluation Metrics

In this section, we focus on evaluation standards for measuring the performance of our custom-trained fine-tuned DL-based YOLOv8s model. For this, we rely on the well-studied fundamental components of the *confusion matrix* from the field of machine learning. These components are *TP* (true positive), *FP* (false positive), *TN* (true negative), and *FN* (false negative). They are used to measure the performance of a model, i.e., how accurately a model distinguishes between the classes. The different combinations and relationships of these components result in other important evaluation metrics, i.e., *precision*, *recall*, *F1 score*, *intersection-over-union* (IoU), *mean average precision* (mAP), etc., which lead us to another level of understanding the holistic view of our model’s reliability and generalization capability.

**Precision.** Precision answers the following basic question: How many positive predictions were *actually correct* from all positive predictions made by the model? To answer this, we rely on precision in Equation (1):

(1) $$\mathrm{Precision}=\frac{\mathrm{TP}}{\mathrm{TP}\;+\;\mathrm{FP}}$$

**Recall.** Recall answers the following basic question: How many actual positive instances were *correctly predicted* by the model from all actual positive instances in the data? To answer this, we rely on recall in Equation (2):

(2) $$\mathrm{Recall}=\frac{\mathrm{TP}}{\mathrm{TP}\;+\;\mathrm{FN}}$$

**F1 score.** The F1 score answers the following basic question: How well does the model *balance* between its precision and recall? To answer this, we rely on the F1 score in Equation (3):

(3) $$F1=2\times \frac{\mathrm{Precision}\times \mathrm{Recall}}{\mathrm{Precision}+\mathrm{Recall}}$$

**Mean average precision (mAP).** mAP answers the following basic question: How consistently accurate are the model’s predictions across different recall levels and all classes? To answer this, we rely on mAP in Equation (4):

(4) $$mAP=\frac{1}{N}\sum_{i=1}^{N}AP_{i}$$

where *N* represents the set of classes, i.e., car, motorcyclist, pedestrian, rickshaw, and truck, and $AP_{i}$ represents the average precision of class *i*. It is worth noting that the AP for each class relies on the *IoU criterion* and its thresholds (a common threshold in object detection is 0.5 (50%)). Hence, *mAP@0.5* evaluates the performance’s DL model at 50% of the detected bounding box overlap to the ground truth bounding box. Now that we have the most important evaluation standards, we present our experimental results and provide a discussion to equip the readers with a comprehensive understanding.

## 4. Results and Discussions

The purpose of conducting a series of experiments is to investigate the behaviors of custom-trained and fine-tuned DL models deployed over different devices (those having GPU and non-GPU capabilities) across different layers of the proposed framework. In this section, we discuss the obtained results of the experiments. We evaluated the performance of object detection tasks at different layers, i.e., vehicle, edge, and cloud. The following uniform setting and configuration are used for the experiments. All experiments were conducted on the same custom dataset (as discussed in Section 2.1) and used the same fine-tuned YOLOv8 model (as discussed in Section 2.1) with configuration, i.e., AdamW optimizer, a batch size of 32, and 200 epochs. In the following, we discuss the findings concluded from the experimental results presented in Table 2.

### 4.1. Model Performance Analysis

The experimental results show that the performance of the model for detection-based perception tasks at each layer either with GPU or non-GPU-based devices resulted in the same performance. It is evident from the results that the performance values of metrics such as precision, recall, mAP@0.5, and mAP@0.5:0.95 are the same for all object classes. For instance, the performance metric values, i.e., precision (0.84), recall (0.90), mAP@0.5 (0.93), and mAP@0.5:0.95 (0.72) for detecting a car object will remain the same regardless of deploying the same model over any type of device and across any layer of the framework. The main reason behind such behavior of the deployed model is its performance in object detection over the same set of data, using the same weights of the fine-tuned YOLOv8s model. Therefore, we conclude that this research addressed the gap in investigating the performance of deployed DL models over low and high-resourceful devices across different layers of the proposed framework. Our findings confirm that a DL model deployed over any device in any layer of the hierarchical framework would not lose performance in terms of accuracy, precision, and recall.

**Figure 4:**
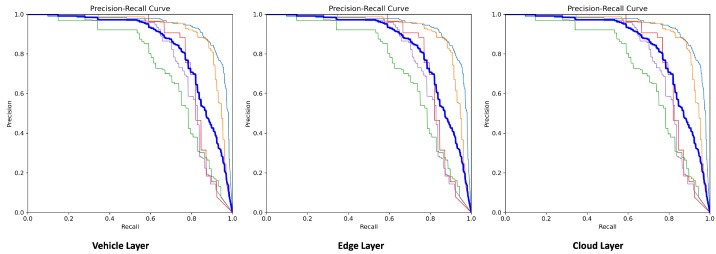
Precision–recall curves at each layer of the framework.

To provide a more comprehensive understanding of the findings, a graphical representation is created to evaluate the DL model performance. Now, we present the precision–recall (PR) curves shown in Figure 4. These curves help in understanding the trade-off between precision and recall for different threshold values. From Figure 4, it should be noted that at each layer, i.e., vehicle, edge, and cloud, the mAP@0.5 is 0.842, which means the deployed YOLOv8s model performed object detection with the same performance. A granular insight can be obtained by looking at the class-level performance of the model, i.e., car (0.93), motorcyclist (0.91), pedestrian (0.73), rickshaw (0.82), and truck (0.79). Hence, we confirm by the graphical representation of PR curves, that the deployment of DL models is not affected by the device types across any layer of the framework.

### 4.2. Time Sensitivity Analysis

The purpose of investigating and comparing time under this section is to find if the detection time is consistent across each layer and any device, regardless of the GPU or non-GPU device type. In Table 2, we observed that the deployed model’s performance is consistent at each layer of the framework. The mAP@0.5 remains constant, regardless of the framework’s layer and device type. Now we show the time comparison of the deployed deep learning model across various devices in Table 3. Since the image capture rate is 30 fps, the average capture time is 33.34 ms. It is important to highlight that—for comparison of time—we rely on the average statistical tool. Therefore, these times are given in the form of averages. Based on the device type, the preprocessing time varies from 1.27 ms to 15.04 ms. The Raspberry Pi device takes a longer time for preprocessing, whereas the Apple M2 Max takes less time to preprocess the spatial data. The lowest preprocessing time for a device in the cloud layer is 2.8 ms.

At the inference time, the prediction is performed from 6.4 ms on a Tesla V100 GPU to 3225.4 ms on a Raspberry Pi 4 CPU. Hence, the Tesla V100 GPU takes less time for inferencing, whereas, less capable devices, like the Raspberry Pi 4 CPU, are slower. The main reason behind this behavior is that Raspberry Pi 4 CPUs are used as gateway devices over vehicles, whereas Tesla V100 GPUs are used as cloud server devices in the cloud layer. Before discussing the total time provided in the table, we look at the transfer time of the images to the edge and cloud layer. Transfer time is relevant for the edge and cloud layers but not for the vehicle layer as the data are captured at the vehicle layer, which eliminates the need for data transfer. When the images are captured at the vehicle layer, they need to be transferred to the edge servers and cloud servers for the deployed models at those layers. Transfer times differ between GPU and non-GPU devices; GPUs have higher upload and download times due to their intensive processing. It is also important to highlight that the images can be captured with the help of a Raspberry Pi 4-associated camera and a Jetson AGX-associated camera at the vehicle layer. Similarly, if we want to capture the images at the edge layer, then the sensory infrastructure of the edge with local sensors will avoid the use of the transfer time by processing the images directly. In any case, the transfer time for both of these device types at the vehicle layer remains zero as we are not required to transfer the data. We directly utilize the data for preprocessing and inferences. However, in the case of edge and cloud layers, we need to upload (transfer) the data. Therefore, we use Google Cloud Storage and connect through the Google API. Using the Google API, we were able to find the upload and download images to the Google Cloud Storage and find the transfer time of the images. To find the total time taken by a deployed model, we rely on the capture, transfer, preprocess, and inference times. The total time taken ranges from 122.9 ms to 3273.8 ms. Our findings confirm that a DL model deployed over any framework’s layer and across any device would consume different times for object detection-based perception of the AVs. In the following, we provide a detailed comparison and discussion of the average preprocess time, average inference time, average transfer time, and average total time for object detection tasks.

**Figure 5:**
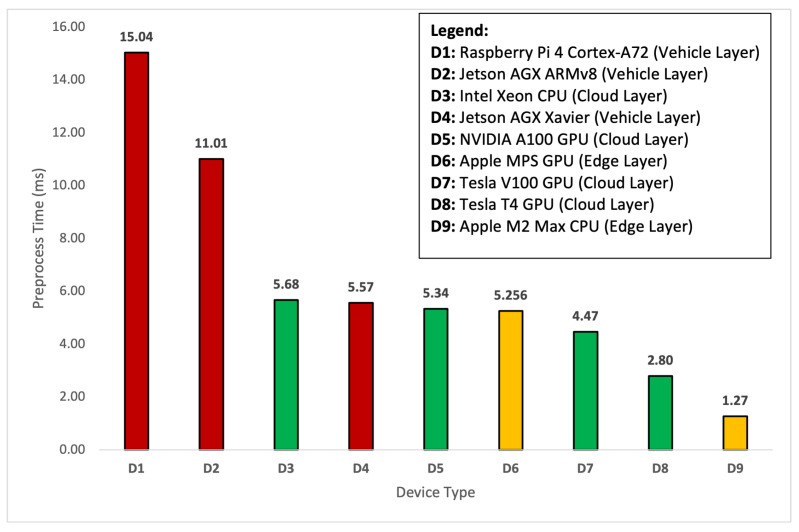
Comparison of the average preprocess time by device type.

To further discuss the results of the preprocessing, the results are presented in graphical form in Figure 5. For the preprocessing, YOLOv8s takes an image, resizes it, and provides padding to make a square shape of the image. Then, the pixel values of the image are normalized using Normalize and converted into a tensor using ToTensor. For resizing and padding, the model utilizes the corresponding Resize and Pad classes. An obvious question arises as to why a DL model needs to preprocess the spatial data when it has already been preprocessed at the data acquisition and pre-processing times, as discussed in Section 2.1. Based on the provided technical descriptions, we justify the preprocessing time taken by our deployed model during the object detection task. This means that, even if we have the spatial dataset in custom-formatted pre-processed form, the DL model would still consume time over the preprocessing stage during the detection process. In Figure 5, the experimental results show the comparison of the average preprocess time taken by various devices at each layer. As it is evident from the figure, the D1 device (Raspberry Pi 4) at the vehicle layer has the highest preprocessing time of 15.04 ms due to low computational resources at the machine. Similarly, the D2 (Jetson AGX ARMv8) device consumes the second-highest preprocessing time of 11.01 ms. For the same reasons, these devices, located at the vehicle layer, consume huge preprocessing times. However, if we look at the devices from D3 to D6 mounted at the vehicle, edge, and cloud layers of the framework, they almost consume the same preprocessing time. Therefore, the results conclude that the preprocessing time can be reduced at the vehicle layer by choosing a GPU-based device. For instance, the D4 (Jetson AGX Xavier) device took the same time as the D5 (NVIDIA A100 GPU) and D6 (Apple MPS GPU) devices mounted at the cloud and edge layers.

**Figure 6:**
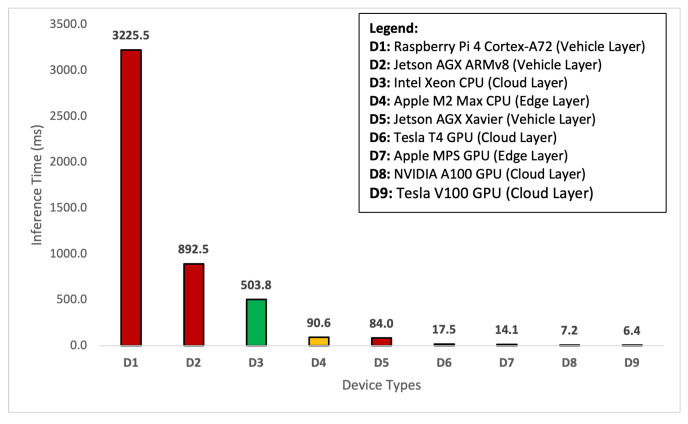
Comparison of the average inference time by device type.

Now that we have analyzed the preprocessing time, we discuss the results of the model inference time shown in Figure 6. The inference time is the time taken by the model to take an image and provide the prediction results. In our case, we focus on object detection tasks in the perception system of the AVs. Therefore, the inference time calculates the detection time from capturing an image to drawing bounding boxes over the objects in the image. Moreover, the inference time plays a vital role in AD. In our case, the AVs need to make real-time object detections while driving on the road to make safe maneuvers. In Figure 6, the experimental results show that inference time is taken by all devices at each layer of the framework. It is evident that D1 (Raspberry Pi 4), a non-GPU-based device, takes the highest inference time of 3225.5 ms followed by D2 (Jetson AGX ARMv8), another non-GPU-based device, which takes 892.5 ms. It is also important to highlight that both of these devices are non-GPU and mounted at the AVs in the vehicle layer. To perform detection using non-GPU devices at the cloud layer, we can rely on devices like D3 (Intel Xeon), which takes almost six times less than that of Raspberry Pi 4 and two times less than Jetson AGX. Furthermore, our findings also confirm the concept of edge computing that if the model is deployed over the edge that is close to the vehicle to detect the object over the road, then the time taken by inference can significantly be reduced. As can be seen, the use of a non-GPU-based edge server, i.e., D4 (Apple M2 Max), can reduce the detection time by five times. On the other hand, if a GPU-based device, i.e., D5 (Jetson AGX Xavier), is available at the vehicle layer, then it will consume an equivalent time in comparison to the edge server. From devices D6 to D9, our experimental results show that GPU-based devices outperform other devices. This means that the GPU configured computational intensive devices are the ideal choice for finding the inferences of the objects.

**Figure 7:**
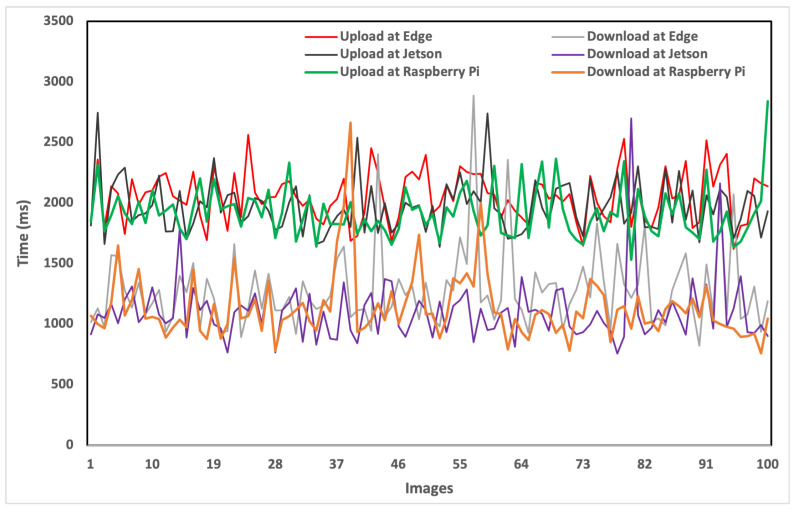
Comparison of transfer time (uploading and downloading of images).

**Figure 8:**
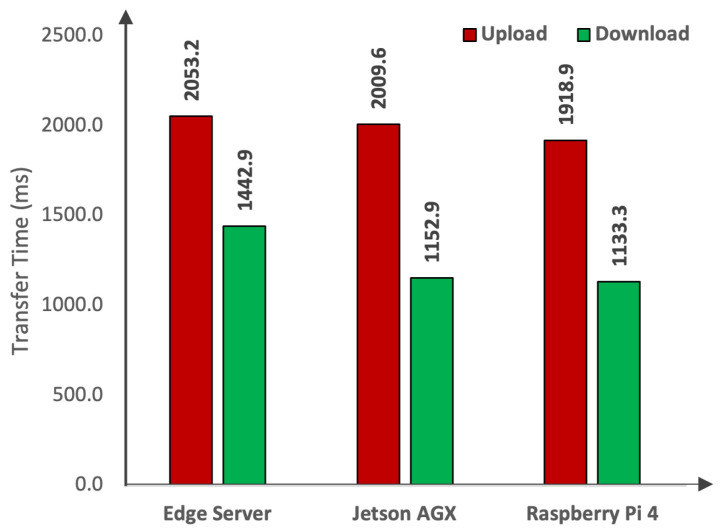
Comparison of average transfer time.

Now that we have analyzed the inference time, we start with the analysis of the average transfer time. The transfer time refers to the time that is taken by either uploading or downloading the spatial data. To perform this set of experiments, we rely on the standard storage available from the Google platform, i.e., Google Cloud Storage. The main reason behind using this storage is the availability of Google Cloud API for upstream and downstream tasks. To understand the working principle of transfer time, let us consider two different scenarios: (1) data captured at the vehicle layer; and (2) data captured at the edge layer. In the first scenario, assume a vehicle with limited computational power to deploy a DL model at the vehicle layer. In this case, the first step is that the vehicle needs to capture the data via a camera sensor associated either with Raspberry Pi 4 or Jetson AGX. After capturing the data via an RPi camera, the vehicle needs to upload the images to the nearest edge device as the edge layer. The upload from Raspberry Pi 4 and download at the edge device occur at different times as shown in Figure 7, which also includes a representation of various other devices and the diverse timing for uploads and downloads for each image in the dataset. The upload time for any device exceeds the download time. Moreover, the variation in times indicates the network fluctuation during the upload and download processes. The average transfer time taken by these devices is shown in Figure 8. Raspberry Pi 4 takes 1918.9 ms to upload the images to the edge server. Similarly, if the data are captured by a Jetson-based camera device, then the Jetson takes 2009.6 ms to upload the images from the vehicle to the edge server. On the other hand, the edge server needs to download the uploaded images from its local cloud for object detection-based perception tasks of the AVs. It is important to note that for a fair comparison, we also utilized the Google Cloud Storage at the edge layer. As can be seen in Figure 8, the edge server takes 1442.9 ms to download the images for the perception task of AVs.

In the second scenario, we consider that the data are captured at the edge layer using a camera sensor mounted on the eRSU. After capturing the data, the eRSU is provided with different options to process the data and perform object detection. The first option is that the object detection can be performed over the edge servers at the edge layer. Therefore, we do not need to transfer the images to other layers of the framework. However, if the edge servers are not capable of deploying the DL models, then we need to transfer the data to other devices either at the vehicle or cloud layer. Therefore, the edge server takes 2053.2 ms to upload images to Google Cloud. The second option is that the vehicle downloads the data from the eRSU. For the vehicle, the download time at Raspberry Pi 4 is 1133.3 ms, and at Jetson AGX, it is 1152.9 ms. Since we considered the same network, the download time for the cloud server will be similar to the download time of the edge server shown in Figure 8. Regardless of where the images are captured, if the underlying layer is not capable of detecting the objects due to limited computation and resources (e.g., the use of non-GPU devices in the vehicle and edge layer), it will upload the images to the cloud for object detection. The upload times can be taken from Figure 8; however, there will be no download time for cloud servers as the cloud will receive the images and perform the object detection within the cloud.

**Figure 9:**
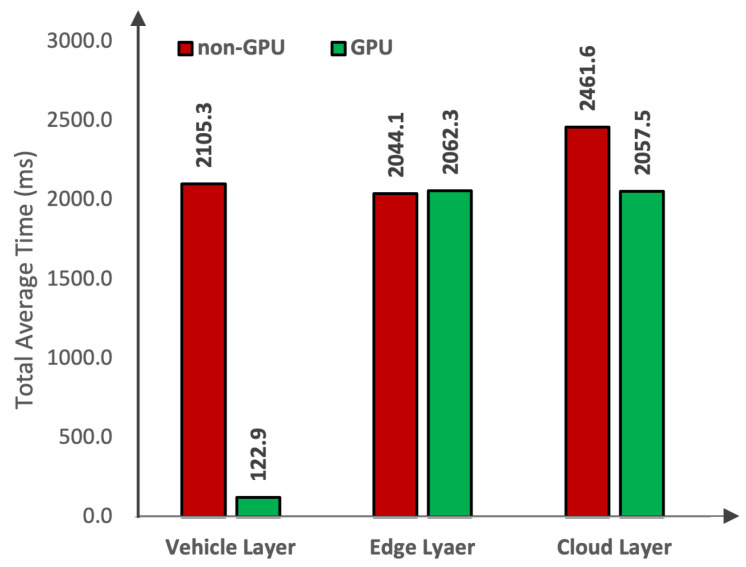
Comparison between the total average times (from capturing the image to inferring the object).

Now that we have analyzed the transfer time, we start with the analysis of the total average time taken by the deployed DL model. The total average time refers to the total time that is taken from capturing the data to detecting the objects in the data. Therefore, to investigate the impact of total average time, we considered non-GPU and GPU-based devices at each layer of the framework as shown in Figure 9. At the vehicle layer, a non-GPU device takes 2105.3 ms to perform object detection. Whereas, a GPU-based device takes 122.9 ms to perform object detection. Therefore, the results conclude that the GPU-based devices outperform non-GPU-based, achieving a 94% reduction in the total time taken for the object detection task. At the edge layer, a non-GPU device takes 2044.1 ms to perform object detection. Whereas, a GPU-based device takes 2062.3 ms to perform object detection. Therefore, we conclude that regardless of any device type at the edge layer, the object detection task consumes the same amount of time. At the cloud layer, a non-GPU device takes 2461.6 ms to perform object detection. Whereas, a GPU-based device takes 2057.5 ms to perform object detection. Therefore, the experimental results conclude that GPU-based devices outperform non-GPU-based devices, achieving a 17% reduction in the total time taken for the object detection task. It is important to highlight that the reason behind the significant reduction in the total average time from capturing the data to inferencing the objects at the vehicle layer is attributed to leveraging gateway devices for capturing the spatial data at the AVs. Moreover, one of the crucial findings of these experiments is that during the utilization of a non-GPU-based device at the vehicle layer, it is recommended to transfer the data to the nearest edge server for object detection tasks. This is because any computational device mounted at the edge layer, i.e., a non-GPU or a GPU-based device, outperforms the total time taken by the gateway device of the vehicle layer.

## 5. Conclusions

The rapid developments in CCAM infrastructures for AVs have achieved significant milestones. In this research paper, we presented a computational framework for analyzing the deployment of a custom-trained fine-tuned DL YOLOv8 model across different devices at the vehicle-edge-cloud layered architecture. We addressed a scientific research gap by investigating the performance of the deployed DL models over low and high-resourceful devices across different layers of the proposed framework. Our results conclude that the GPU-based devices outperform non-GPU-based devices, achieving a 94% reduction in the total average time taken for the object detection task at the vehicle layer. In addition, we confirmed that the choice of the device type is not important for the deployment of DL models at the edge layer. Moreover, our experimental results showed that GPU-based devices outperform non-GPU-based devices, achieving a 17% reduction in the total average time taken for the object detection task at the cloud layer of the framework. The findings of our research work may help the relevant research community in finding less computationally demanding devices suitable for specific levels from a vehicle-edge-cloud-layered framework.

## Figures and Tables

**Table 1:** The experimental setup for various devices at different layers.

Layers	Platforms	Devices	Device Type	Memory
**Cloud Layer**	Google Cloud	Tesla V100	GPU	16 GB
	Google Cloud	NVIDIA A100	GPU	40 GB
	Google Cloud	Tesla T4	GPU	15 GB
	Google Cloud	Intel Xeon	non-GPU	16 GB
**Edge Layer**	Apple	MPS	GPU	32 GB
	Apple	M2 Max	non-GPU	32 GB
**Vehicle Layer**	Jetson AGX	Xavier	GPU	16 GB
	Jetson AGX	ARMv8	non-GPU	16 GB
	Raspberry Pi 4	Cortex-A72	non-GPU	08 GB

**Table 2:** Performance metrics for the fine-tuned deep learning model on different devices.

Layers	Devices	Objects	Precision	Recall	mAP@0.5	mAP@0.5:0.95
Cloud Layer Edge Layer Vehicle Layer	GPUs and non-GPUs	car	0.842	0.901	0.938	0.724
		motorcyclist	0.868	0.877	0.916	0.655
		pedestrian	0.700	0.693	0.739	0.430
		rickshaw	0.881	0.759	0.826	0.614
		truck	0.783	0.716	0.794	0.583

**Note:** At each layer, the same fine-tuned deep learning model is deployed for both GPU and non-GPU devices with the following parameters. Dataset: custom-formatted; model: YOLOv8s; optimizer: AdamW; batch size: 32; epochs: 200.

**Table 3:** Time comparison of the deployed deep learning model over different devices.

Layers	Platforms	Devices	Capture	Transfer	Preprocess	Inference	Total
Cloud Layer	Google	Tesla V100	33.34	2009.56	4.47	6.401	2053.77
	Google	NVIDIA A100	33.34	2009.56	5.34	7.223	2055.46
	Google	Tesla T4	33.34	2009.56	2.80	17.456	2063.16
	Google	Intel Xeon	33.34	1918.86	5.68	503.75	2461.63
Edge Layer	Apple	MPS	33.34	2009.56	5.256	14.108	2062.26
	Apple	M2 Max	33.34	1918.86	1.27	90.60	2044.08
Vehicle Layer	Jetson AGX	Xavier	33.34	0	5.57	84.03	122.94
	Jetson AGX	ARMv8	33.34	0	11.01	892.51	936.86
	Raspberry Pi	Cortex-A72	33.34	0	15.04	3225.46	3273.84

## Data Availability

The custom road dataset is available at Zenodo (https://zenodo.org/records/10610036) (accessed on 2 February 2024).
